# Supplementary material for: DMRforPairs: identifying Differentially Methylated Regions between unique samples using array based methylation profiles
Source: BMC Bioinformatics. 2014 May 15;15:141. doi: 10.1186/1471-2105-15-141 (PMC4046028; doi:10.1186/1471-2105-15-141)
Supplement: Additional file 2 — DMRforPairs output for the comparison of A431-MCF7 and NA17018-NA17105. Please start from the HTML files in each folder. Available via the BMC Bioinformatics website. [file 1471-2105-15-141-S2.zip › 1394847754114233_MOESM2_ESM/A431_MCF7/figures/31392.pdf]

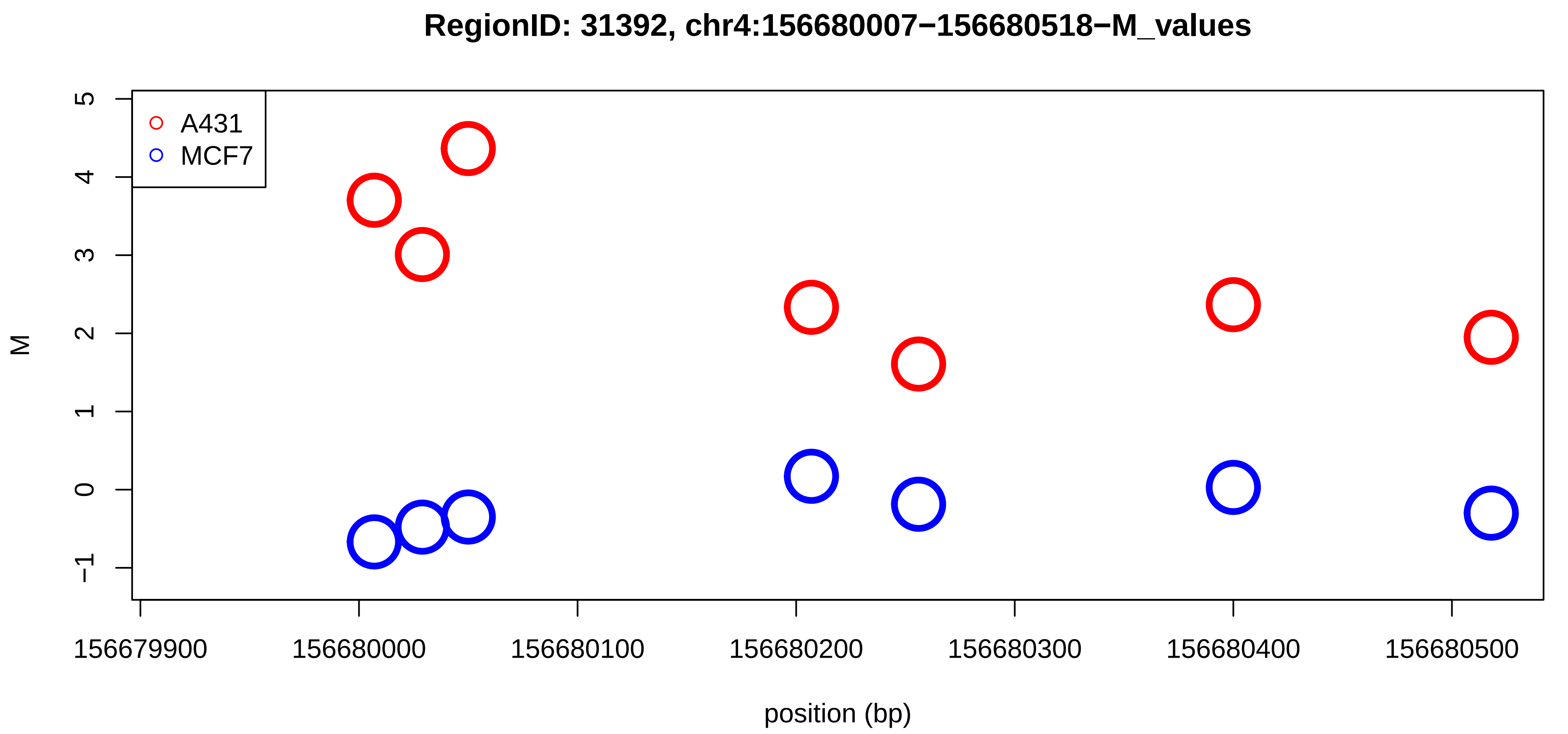
RegionID: 31392, chr4:156680007−156680518−M_values
A431
MCF7
M
5
4
3
2
1
0
−1
156679900
156680000
156680100
156680200
156680300
156680400
156680500
position (bp)

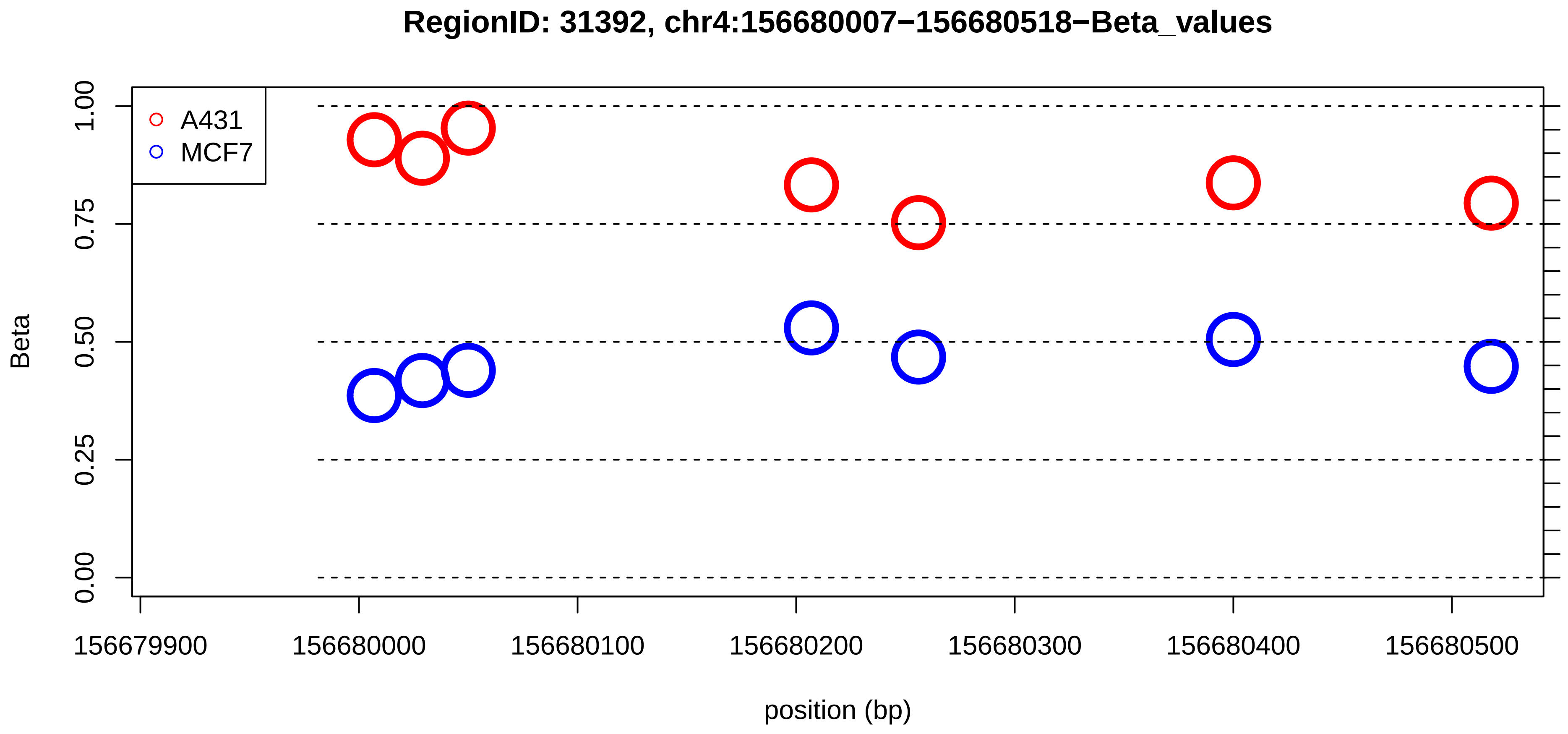
RegionID: 31392, chr4:156680007−156680518−Beta_values
A431
MCF7
Beta
1.00
0.75
0.50
0.25
0.00
156679900
156680000
156680100
156680200
156680300
156680400
156680500
position (bp)

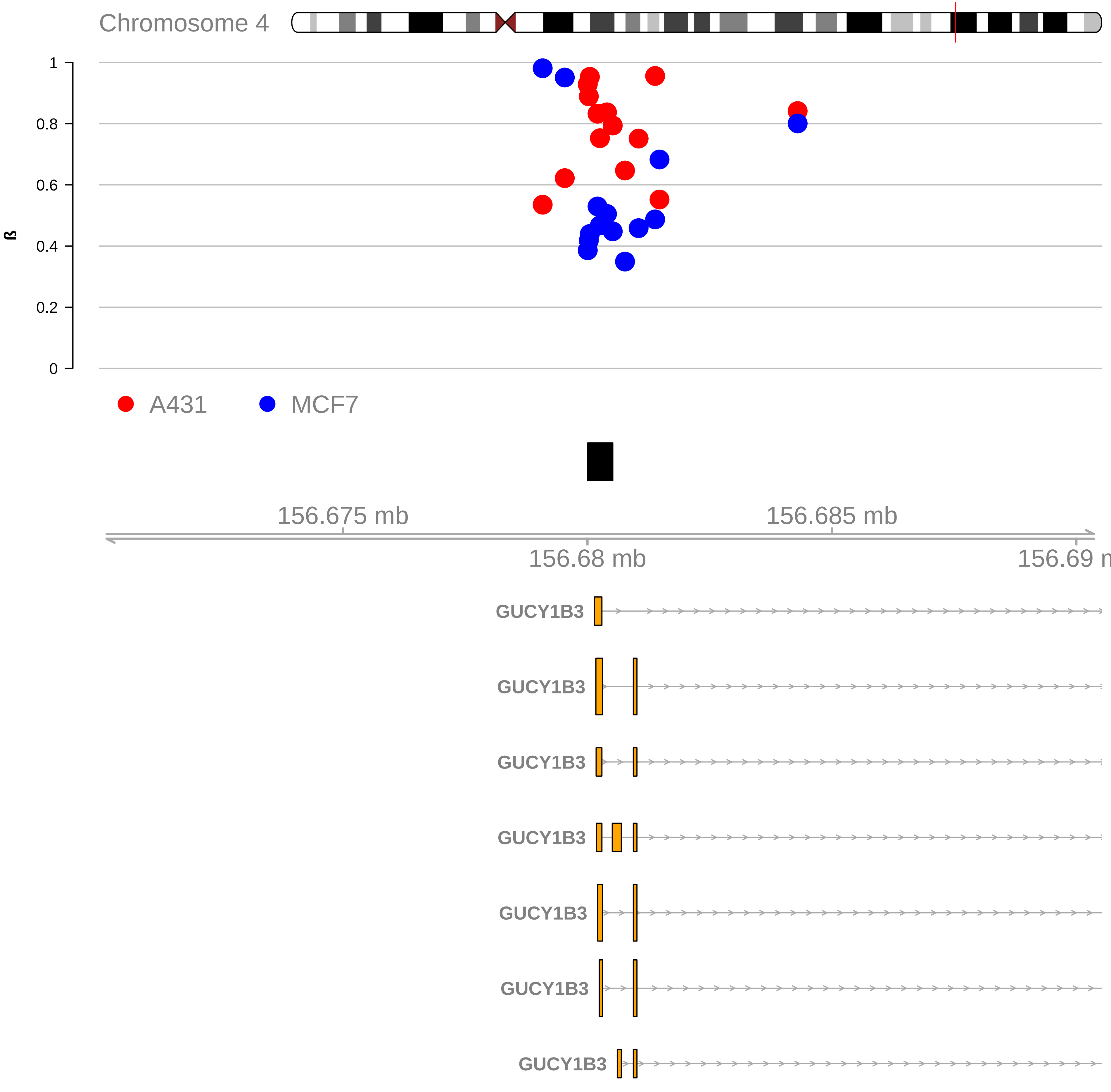

Chromosome 4
ß
1
0.8
0.6
0.4
0.2
0
A431
MCF7
156.675 mb
156.685 mb
156.68 mb
156.69 m
GUCY1B3
GUCY1B3
GUCY1B3
GUCY1B3
GUCY1B3
GUCY1B3
GUCY1B3
